# Anti-mycobacterium tuberculosis activity of polyherbal medicines used for the treatment of tuberculosis in Eastern Cape, South Africa

**DOI:** 10.4314/ahs.v17i3.21

**Published:** 2017-09

**Authors:** Elizabeth B Famewo, Anna M Clarke, Ian Wiid, Andile Ngwane, Paul van Helden, Anthony J Afolayan

**Affiliations:** 1 Faculty of Science and Agriculture, University of Fort Hare, Alice 5700, South Africa; 2 DST-NRF Centre of Excellence for Biomedical Tuberculosis Research, SAMRC Centre for Tuberculosis Research, Division of Molecular Biology and Human Genetics, Faculty of Medicine and Health Sciences, Stellenbosch University, PO Box 241, Cape Town 8000, South Africa

**Keywords:** *Mycobacterium tuberculosis*, in vitro activity, polyherbal medicines, South Africa

## Abstract

**Background:**

The emergence of drug-resistant strains of *Mycobacterium tuberculosis* has become a global public health problem. Polyherbal medicines offer great hope for developing alternative drugs for the treatment of tuberculosis.

**Objective:**

To evaluate the anti-tubercular activity of polyherbal medicines used for the treatment of tuberculosis.

**Methods:**

The remedies were screened against *Mycobacterium tuberculosis* H37Rv using Middlebrook 7H9 media and MGIT BACTEC 960 system. They were liquid preparations from King Williams Town site A (KWTa), King Williams Town site B (KWTb), King Williams Town site C (KWTc), Hogsback first site (HBfs), Hogsback second site (HBss), Hogsback third site (HBts), East London (EL), Alice (AL) and Fort Beaufort (FB).

**Results:**

The susceptibility testing revealed that all the remedies contain anti-tubercular activity with KWTa, KWTb, KWTc, HBfs, HBts, AL and FB exhibiting more activity at a concentration below 25 µl/ml. Furthermore, MIC values exhibited inhibitory activity with the most active remedies from KWTa, HBfs and HBts at 1.562 µg/ml. However, isoniazid showed more inhibitory activity against *M. tuberculosis* at 0.05 µg/ml when compare to the polyherbal remedies.

**Conclusion:**

This study has indicated that these remedies could be potential sources of new anti-mycobacterial agents against *M. tuberculosis*. However, the activity of these preparations and their active principles still require in vivo study in order to assess their future as new anti-tuberculosis agents.

## Introduction

*Mycobacterium tuberculosis*, the leading causative agent of tuberculosis (TB) is responsible for the morbidity and mortality of a large population worldwide^1^. TB has a long co-evolutionary history with humans. It does not exhibit any symptom of disease except when impairment of immunity arises due to malnutrition, diabetes, malignancy and AIDS^2^; however, about 10% of healthy individuals may develop active TB in their life time due to genetic factors. The ability of TB to resist drugs and the influence of HIV epidemic has made the disease remain a devastating global public health problem^3^. According to WHO^4^, one-third of the world's population have been infected with *Mycobacterium tuberculosis* (MTB). In 2014, an estimated number of 9.6 million new TB infections were reported, of which 5.4 million were men; 3.2 million were women and 1.0 million children^3^. This disease is responsible for approximately two million deaths annually^5^.

Some of the main obstacles to the global control of the disease are the HIV epidemic that has dramatically increased the risk of developing active TB, increasing emergence of multidrug resistant-TB (MDR-TB: resistance to isoniazid and rifampin) and refractory nature of latent TB treatment to conventional anti-TB drugs^6^,^7^,^8^,^9^. The situation is further exacerbated by the increasing development of extensively drug-resistant (resistant to MDR-TB, all fluoroquinolones and at least one of the second-line anti-TB injectable drugs including amikacin, kanamycin and/or capreomycin)^10^,^11^. According to the modes of action of these drugs, they can be grouped as cell wall inhibitors (isoniazid, ethambutol, ethionamide, cycloserine), nucleic acid synthesis inhibitors (rifampicin and quinolones), protein synthesis inhibitors (streptomycin, kanamycin) and inhibitors of membrane energy metabolism (pyrazinamide)^12^,^13^,^14^. For instance, Isoniazid (INH )is the most widely used treatment for TB and its latent infections^15^. This drug enters the cell as a pro-drug, which is activated by MTB catalase-peroxidase enzyme (KatG). The enzyme activates INH and facilitates its interaction with various toxic reactive species (oxides, hydroxyl radicals and organic moieties) in the bacterial cell^16^, thereby, weakening the components of the cell wall and finally, the death of the bacteria^17^. INH targets inhA enzyme (enoylacyl carrier protein reductase), which is involved in the elongation of fatty acids in mycolic acid synthesis^18^. The replacement of an amino acid in the NADH binding site of inhA results into INH resistance, preventing the inhibition of mycolic acid biosynthesis^19^. INH-resistant strains often lose catalase and peroxidase activities due to KatG Ser315Thr mutation^20^. Resistance to INH can also occur through mutations in the promoter region of inhA, leading to over expression of inhA, or by mutations at the inhA active site, thereby lowering inhA affinity for INH^21^. Rifampicin (RIF) have been used as first-line drug in combination with other therapies for the treatment of TB infections. RIF is believed to inhibit bacterial DNA-dependent RNA polymerase^9^. This drug interferes with RNA synthesis by binding to the β subunit of *mycobacterial* RNA polymerase, which is encoded by rpoB, thereby killing the organism. Resistance to RIF arises due to missense mutations in the gene. Mtb resistance to RIF occurs at a frequency of 10−7 to 10−8 as a result of mutations in rpoB^22^. About 96% of all mutations are found in the 81-bp core region of the gene between codons 507 and 533, with the most common changes occurring in codons Ser531Leu,His526Tyr and Asp516Val^23^.

Pyrazinamide (PZA) is another vital first-line drug used for the treatment of TB. It plays an important role in reducing the duration of TB treatment^24^. PZA is a pro-drug that requires conversion to its active form, pyrazinoic acid (POA) by the *mycobacterial* enzyme pyrazinamidase/nicotinamidase. The efflux system of the mycobacterial cell enables massive accumulation of POA in the bacterial cytoplasm, leading to disruption of the bacterial membrane potential^25^,^26^. The exact mechanism of PZA resistance remains unknown^9^. However, PZA resistance has been associated with defective pyrazinamidase/nicotinamidase activity which results from mutations that might occur at different regions (3–17, 61–85 and 132–142) of pyrazinamidase/nicotinamidase^27^.

Ethambutol (EMB) is a first-line drug used in combination with INH, RIF and PZA preventing the emergence of drug resistance *mycobacterium*. This drug interferes with the cell wall of MTB through a synthetic mechanism thereby inhibiting arabinosyl-transferase (embB), an enzyme involved in cell wall biosynthesis28. The enzyme has been proposed as the target of EMB in Mtb11. Mutation is the cause of EMB resistance and it occurs at a rate of approximately 1 in 107 organisms. It increases the production of arabinosyl-transferase, which overwhelms the inhibitory effects of EMB. Studies have revealed five mutations in codon 306 accounting 70–90% of all EMB resistant strains^29^. The resistance of Mtb to TB-drugs is mostly due to mutation which is a cause for concern. Therefore, it is important to search for new anti-*tuberculosis* agents, preferably those that can be readily and simply produced from medicinal plants.

It has been estimated that about 80% of South African population is infected with tuberculosis, with 88% highest prevalence of latent TB among the age group of 30–39 years old living in the rural settlements^30^. However, the strains of drug resistant tuberculosis have been on increase yearly in the country^31^.

Polyherbal remedies have been used extensively for the treatment of various diseases for many centuries. They are mixtures of various herbs which contain multiple active constituents and act synergistically against infections^32^. Natural products and/or their semi-synthetic derivatives are important sources of new chemical compounds that might play an important role in the chemotherapy of tuberculosis^33^. Several studies on the use of polyherbal medicines have revealed that these therapies possess pharmacological functions. For instance, *Rajanyamalakadi*, a polyherbal preparation which contains three herbal ingredients has been proven to show significant anti-diabetic, hypolipidemic and anti-oxidant properties^34^. Also, Polyherbal health tonic tea used for the treatment of an array of diseases affecting humans and Sanjivani Vati used for the treatment of cough and cold have been shown to possess significant pharmacological activities^35^,^36^. Other Polyherbal remedies such as *Livina, Rhumapar* tablet, *Diakyur* and Sugar Remedy have been proven to contain pharmacological activities^37^,^38^,^39^,^40^.

Many researchers have reported on the inhibitory properties of medicinal plants against *Mycobacterium tuberculosis* both in South Africa and in other countries^33^,^41^,^42^ but there is a dearth of information on the inhibitory properties of polyherbal medicines against this organism. The aim of the present study therefore was to evaluate polyherbal remedies used for the treatment of TB for anti-*Mycobacterium tuberculosis* activities.

## Materials and methods

### Collection of polyherbal medicines

**Figure 1:**
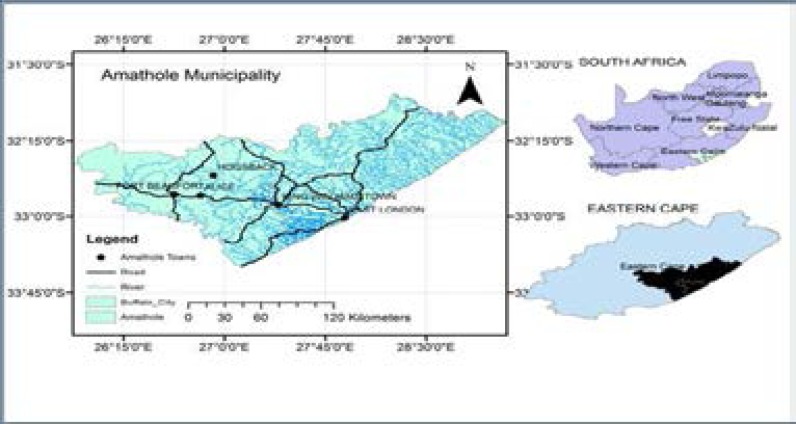
Map of Amathole District Municipality^43^

A total of nine polyherbal medicines evaluated in this study were purchased from herbal sellers in five communities namely; Alice, Fort Beaufort, Hogsback, King Williams Town and East London in Amathole District Municipality of the Eastern Cape Province, South Africa (Figure 1). Each remedy was labelled and coded according to the place of collection; viz: King Williams Town site A (KWTa), King Williams Town site B (KWTb), King Williams Town site C (KWTc), Hogsback first site (HBfs), Hogsback second site (HBss), Hogsback third site (HBts), East London (EL), Alice (AL) and Fort Beaufort (FB). The small number of remedies obtained in this study was due to the fact that only a few traditional healers treat and sell remedies for TB. They claim to have acquired the knowledge from their ancestors; and this knowledge is been transferred from one generation to another. The herbal ingredients present in each of the remedies are shown in Table 1. The remedies were already prepared with water by the herbal sellers into clean 2-litre containers. They were then transported to Medicinal Plants and Economic Development Research Centre, University of Fort Hare for analysis.

**Table 1:** Herbal ingredients present in each of the polyherbal medicines used for the treatment of tuberculosis in Amathole district municipality,

Name/code	Local name	Botanical name	Parts used
AL	Mountain garlic Mlomo mnandi Red carrot Inongwe Mnonono River pumpkin Herbal menthol leaf Herbal buchu water	*Allium sativum* (L.) *Glycyrrhiza glabra* (L.) *Daucus carota* (L.) *Hypoxis argentea* (Fiscand) *Strychnos decussata* (Pappe) Gilg *Gunnera perpensa* (L.) *Mentha piperita* (L.) *Agathosma betulina* (Berg)	Rhizome Root Root corms Bark Rhizome Leaf Leaf
EL	Inongwe Intelezi Ngcambumvuthuza Inqwebeba Iqwili	*Hypoxis argentea* (Fiscand) *Haworthia reinwardtii* (Haw) *Ranunculusmultifidus* (Forssk) *Albuca flaccid* (Jacq.) *Alepidea amatymbica* (Eckl. & Zeyh.)	corms Leaf Root Leaf Rhizome
FB	Buchu leaf Mountain garlic Ginger Chilli pepper	*Agathosma betulina* (Berg) *Allium sativum* (L.) *Zingiber officinalis* (L.) *Capsicum annuum* (L.)	Leaf Rhizome Rhizome Fruit
KWTa	Maphipha Mnonono Ixonya Inongwe Sicimamlilo Iphuzi	*Rapanea melanophloeos (*L.) *Strychnos decussate* (Pappe) Gilg *Kniphofia drepanophylla* (Baker) *Hypoxis argentea* (Fiscand) *Pentanisia prunelloides* (Klotzsch) *Centella eriantha* (Rich.)	Bark Bark Root Corms Rhizome Rhizome
KWTb	Umdlavuza Mnonono Inceba emhlophe	*Lauridiatetragonia* (L.F.) *Strychnos decussate* (Pappe) Gilg *Hermannia* sp. (L.)	Root Bark Root
**Name/code**	**Local name**	**Botanical name**	**Parts used**
KWTc	Mnonono	*Strychnos decussate* (Pappe)	Gilg Bark
HBfs	Red carrot Mlungu mabele Calmoes Mountain garlic	*Daucus carota* (L.) *Zanthoxylum capense* (Thunb.) *Acorus calamus (*L.) *Allium sativum* (L.)	Root Bark Rhizome Rhizome
HBss	Buchu leaf Chilli pepper	*Agathosma betulina* (Berg) *Capsicum annuum* (L.)	Leaf Vegetable
HBts	Maphipha Red carrot Uroselina Mountain garlic	*Rapanea melanophloeos* (L) Mez *Daucus carota* (L.) *Cinnamomum camphora* (L.) J. Presl *Allium sativum* (L.)	Bark Root crop Bark Rhizome

### Sample preparation

The already prepared water remedies were put in 2-liter containers. Each remedy was filtered with a Buchner funnel and Whatman No. 1 filter paper. The filtrate obtained was frozen at −40°C and freeze dried for 48h using a freeze dryer (Vir-Tis benchtop K, Vir-Tis Co., Gardiner, NY). The resulting sample was dissolved in 100% dimethylsulfoxide (DMSO) to a concentration of 50 mg/ml to make a stock solution^45^.

### Microbial strain and medium used for the assays

Reference MTB strain H37Rv (ATCC 25618) was used for the anti-*Mycobacterium tuberculosis* assay. It was obtained from American Type, MD, USA Culture Collection. Bacterial culture with DMSO (1.2%), isoniazid (INH) at MIC99 (0.05 µg/ml) and bacterial culture only were used as controls^46^.

### Bacterial culture and drug preparation

Suspensions of *Mycobacterium tuberculosis* H37Rv were grown using *mycobacterial* growth indicator tubes (MGIT). The inocula were prepared from Lowenstein-Jensen slants. To prepare an inoculum that was less than 15 days old from a culture grown on Lowenstein-Jensen medium, a suspension was prepared in saline and adjusted to a 1.0 McFarland standard. The suspension was vortexed for several minutes and was allowed to stand for 20 min for the initial settling of larger particles. The supernatant was transferred to an empty sterile tube and was allowed to stand for an additional 15 min. After being transferred to a new sterile tube, it was then adjusted to a 0.5 McFarland turbidity standard by visual comparison. A 1:5 dilution of the bacterial suspension was prepared, and 0.5 ml was inoculated into MGIT 7H12® (MGIT 960 system, Becton Dickinson, Sparks, USA) tubes containing test and control compounds^46^.

The growth of the organism was monitored through fluorescent changes due to oxygen consumption in the medium during active growth. Aliquots (100 µl) of each herbal medicine was added to the MGIT tubes containing bacteria in Middlebrook 7H12® media, with the final DMSO concentration not exceeding 1.2%. The tubes were incubated at 37°C in MGIT system, and growth units (GU) were monitored for six days. All the remedies were tested at concentrations of 50 and 25 ug/ml^46^.

For MIC99 evaluations, a 1% bacterial control culture was prepared in a drug-free MGIT tube and the MIC_99_ of the compound determined relative to the growth units of the control (GU–400). The MIC was determined as the lowest drug concentration that equals or lower than GU of the 1% bacterial culture. Controls that were also included are bacterial culture with DMSO (1.2%), isoniazid (INH) and bacterial culture only. All the herbal preparations were tested at two-fold decreasing concentration^46^.

## Results

In the present study, the susceptibility and minimum inhibitory concentration (MIC) of nine polyherbal medicines were determined against *M. tuberculosis* H37Rv, in vitro. The susceptibility testing revealed that all the remedies have anti-tubercular activity against *M. tuberculosis* H37Rv at concentrations below 50 ug/ml. Seven of these polyherbal preparations, namely; KWTa, KWTb, KWTc, HBfs, HBts, AL and FB showed activity at concentrations below 25 ug/ml, with the remaining remedies showing activity at concentrations between 25 and 50 ug/ml (Table 2).

**Table 2:** Susceptibility testing and minimum inhibition concentration (MIC_99_) of nine polyherbal remedies against *M. tuberculosis* H37Rv using MGIT BACTEC 960 system

Polyherbal remedies	Susceptibility activity (µg/ml)	MIC_99_ of the remedies (µg/ml)
KWTa	< 25	< 1.562
KWTb	< 25	25
KWTc	< 25	25
HBfs	< 25	< 1.562
HBss	> 25	25
HBts	< 25	< 1.562
AL	< 25	3.125
EL	> 25	25
FB	< 25	25
Isoniazid (INH)	-	0.05

All the remedies exhibited inhibitory activity against *M. tuberculosis* H37Rv with KWTa, HBfs and HBts as the most active remedies at 1.562 µg/ml, followed by AL remedy which showed growth inhibition at 3.125 µg/ml. The remaining preparations from KWTb, KWTc, HBss, EL and FB showed growth inhibition against *M. tuberculosis* at 25 µg/ml. However, isoniazid showed more inhibitory activity against *M. tuberculosis* H37Rv at 0.05 µg/ml when compared to the polyherbal remedies (Table 2).

## Discussion

Tuberculosis has been a major health problem for developing countries including South Africa. The increasing resistance of the disease to first and second line drugs has demanded the need for a new search for anti-*mycobacterial* agents that could be effective, efficient, non-toxic and cost effective^47^.

The herbal preparations from KWTa, HBfs, HBts and AL showed a greater anti-*mycobacterial* activity, resulting in lower susceptibility patterns and MIC values observed. From observation, the aforementioned remedies contain a mixture of two or more of the following herbs: *Allium sativum, Strychnos decussata, Daucus carota, Hypoxis argentea, Rapanea melanophloeo* together with other herbs. Species of these plants have been investigated and shown to contain anthraquinones, glycosides, saponins, tannins, terpenoids, aloin, saponins, steroids and flavonoids^48^,^49^,^50^. Other compounds include alkaloids, terpenes, resin, monoterpenoids, sesquiterpenoids and phenols which show activity against *Mycobacterium tuberculosis*^51^,^15^,^52^. *Allium sativum* is a plant that has been reported as an established remedy for the treatment of tuberculosis^53^. It possesses variety of biological properties such as anti-cancer, anti-microbial, antioxidant, immunomodulatory, anti-inflammatory, hypoglycaemic and anti-cardiovascular properties^54^. Several studies conducted on the in vitro activity of *Allium sativum* against *Mycobacterium tuberculosis* revealed that this plant possesses anti-tubercular properties^41^,^42^,^53^,^53^. The presence of sulphur compounds such as allicin, ajoene, allylmethyltrisulfide, diallyltrisulfide, diallyldisulphide has been associated with the anti-tubercular activity of this *Allium sativum*^55^.

Information on the use of *Strychnos decussate* as an anti-tubercular agent has not been reported. This study is the first to report the use of this plant as a remedy for the treatment of TB. However, it has been reported to possess anti-fungal activity^56^. *Daucus carota* is a root vegetable. There are only a few reports on the anti-tubercular activity of this plant^57^,^58^. However, it has been reported to be used as an anti-bacterial^59^, anti-fertility^60^, anti-oxidant^61^, ophthalmic and stimulant^62^, anti-septic, diuretic, hepatoprotective, anti-inflammatory^63^,^64^, anti-helmintic, carminative^65^, deobstruent, diuretic and galactogogue. According to the reports, phenolics, polyacetylenes, carotenoids, ascorbic acid and tocopherol are the most abundant phytonutrients present in this plant^66^. *Hypoxis argentea* has also been reported to be used as a remedy for the treatment of TB^58^. Species of the genus *Hypoxis* have been used as anti-bacterial, anti-fungal, anti-viral, anti-oxidant, anti-inflammatory, anti-diabetic, cardiovascular, anti-convulsant and anti-cancer^67^,^68^,^69^,^70^,^71^. The presence of several compounds, especially glucosides, sterols and sterolins could be responsible for the different activities found in *Hypoxis*^72^. *Rapanea melanophloeo* has been screened for activity and found active against drug-resistant and drug-sensitive strains of *Mycobacterium tuberculosis*^73^,^74^. This plant has been reported to contain bioactive compounds such as benzoquinones, saponins and tannins which could probably contribute to its activity^73^.

The high activity of these polyherbal remedies against *M. tuberculosis* could be attributed to the presence of multiple active constituents which may act in synergy and produce greater anti-*mycobacterial* activity. This is an indication that many natural products are potential source of antimycobacterial agents^42^.

## Conclusion

This study has revealed that polyherbal remedies have the potential to cure tuberculosis. This is the first research work on the anti-*tuberculosis* activity of polyherbal medicines used for the treatment of tuberculosis in South Africa. The remedies might be potential sources of new anti-mycobacterial agents as they all showed activity against *M. tuberculosis*. However, the activity of these remedies and their active principles still require in vivo study in order to validate their potential as anti-*tuberculosis* agents.
